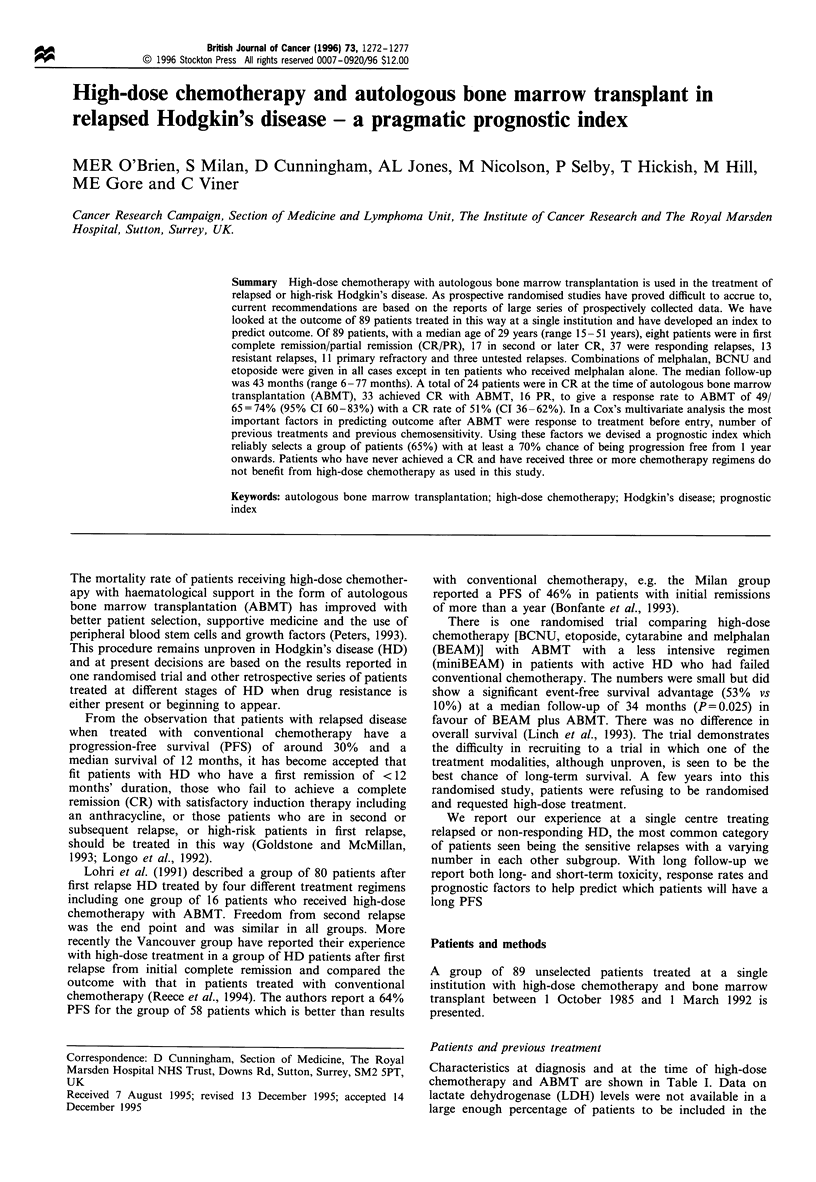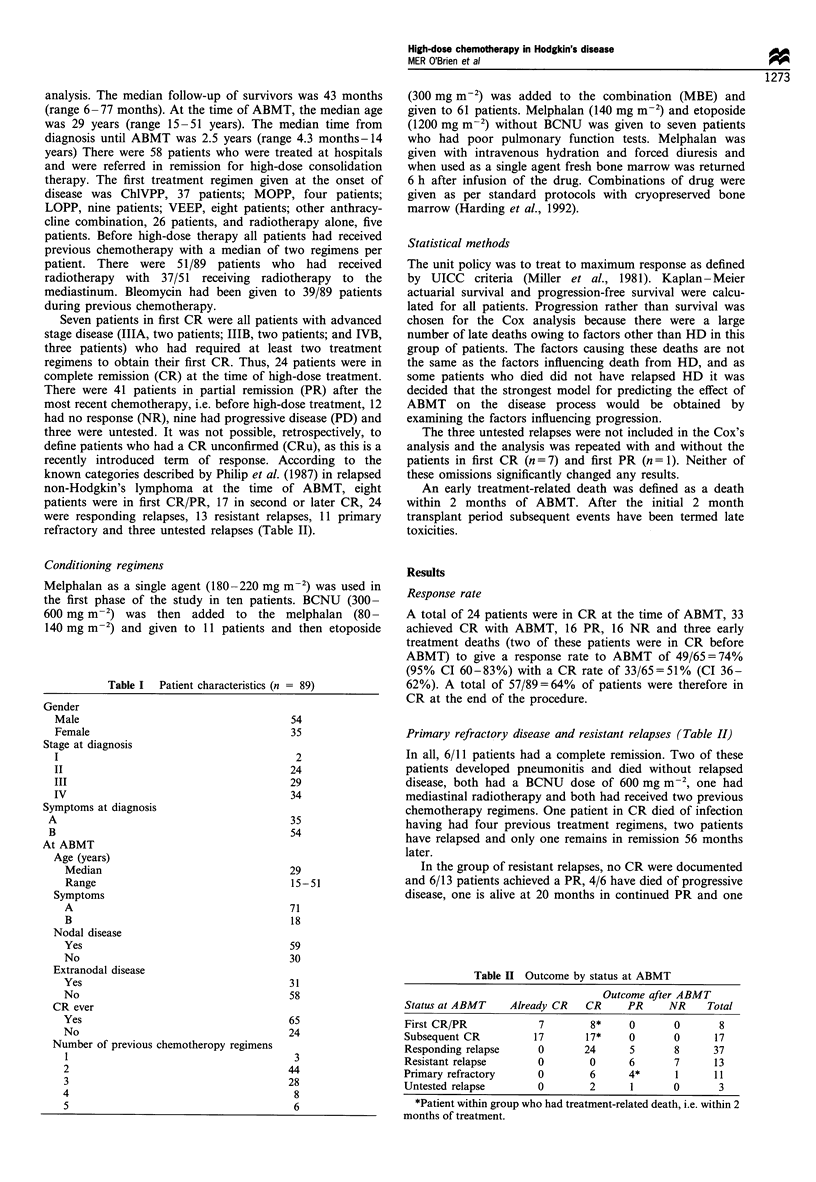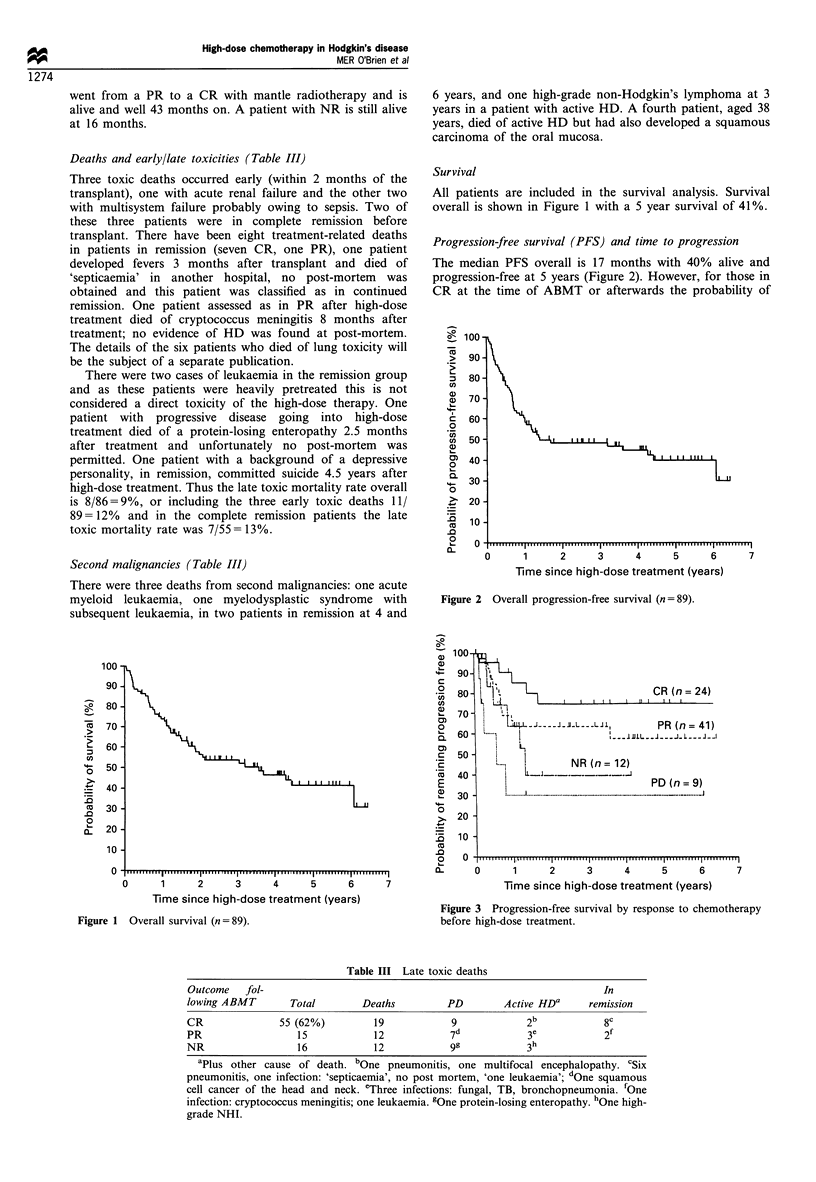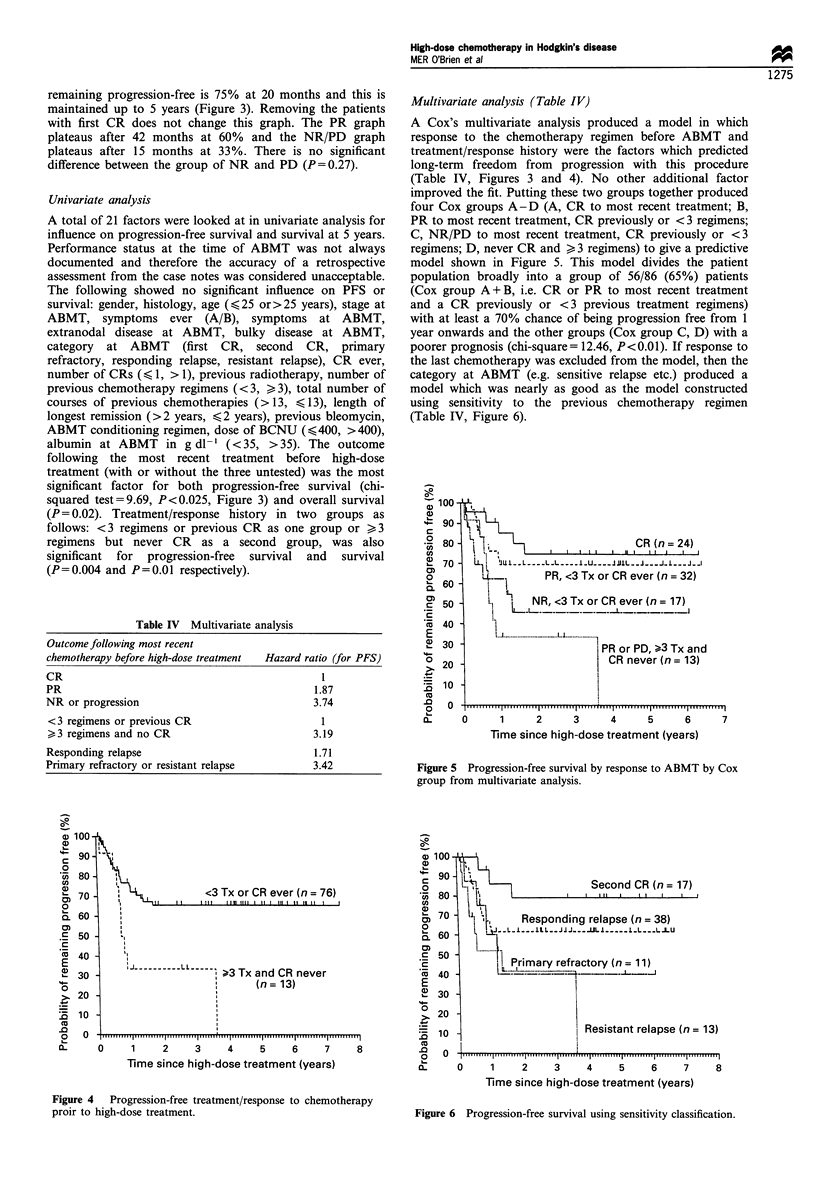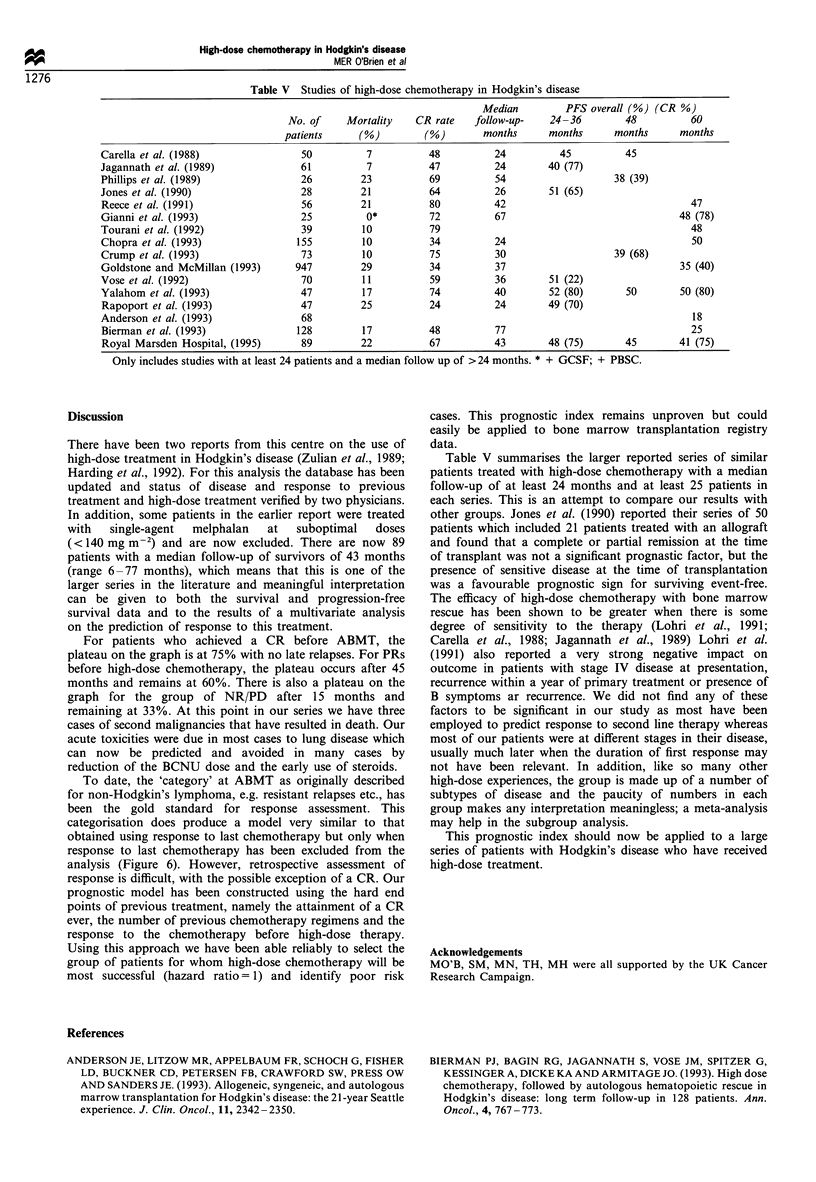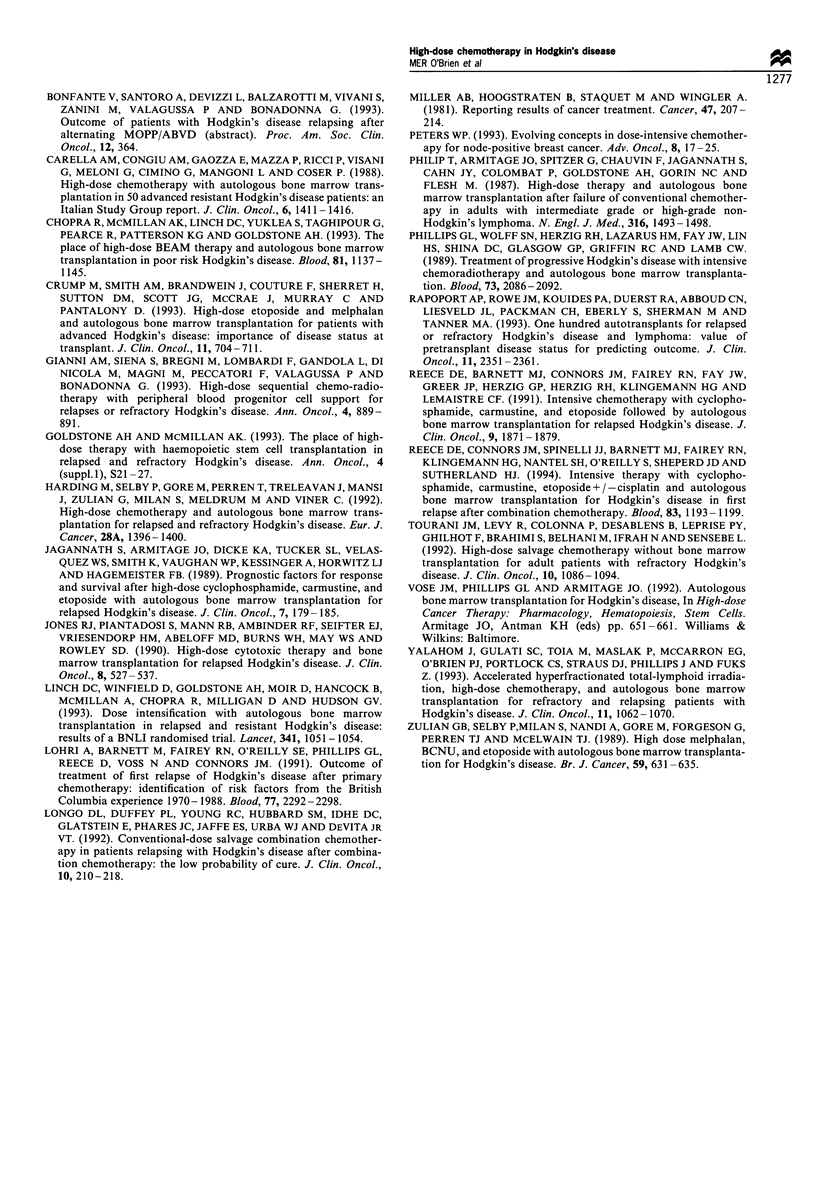# High-dose chemotherapy and autologous bone marrow transplant in relapsed Hodgkin's disease--a pragmatic prognostic index.

**DOI:** 10.1038/bjc.1996.244

**Published:** 1996-05

**Authors:** M. E. O'Brien, S. Milan, D. Cunningham, A. L. Jones, M. Nicolson, P. Selby, T. Hickish, M. Hill, M. E. Gore, C. Viner

**Affiliations:** Cancer Research Campaign, Section of Medicine, Sutton, Surrey, UK.

## Abstract

High-dose chemotherapy with autologous bone marrow transplantation is used in the treatment of relapsed or high-risk Hodgkin's disease. As prospective randomised studies have proved difficult to accrue to, current recommendations are based on the reports of large series of prospectively collected data. We have looked at the outcome of 89 patients treated in this way at a single institution and have developed an index to predict outcome. Of 89 patients, with a median age of 29 years (range 15-51 years), eight patients were in first complete remission/partial remission (CR/PR), 17 in second or later CR, 37 were responding relapses, 13 resistant relapses, 11 primary refractory and three untested relapses. Combinations of melphalan, BCNU and etoposide were given in all cases except in ten patients who received melphalan alone. The median follow-up was 43 months (range 6-77 months). A total of 24 patients were in CR at the time of autologous bone marrow transplantation (ABMT), 33 achieved CR with ABMT, 16 PR, to give a response rate to ABMT of 49/65 = 74% (95% CI 60-83%) with a CR rate of 51% (CI 36-62%). In a Cox's multivariate analysis the most important factors in predicting outcome after ABMT were response to treatment before entry, number of previous treatments and previous chemosensitivity. Using these factors we devised a prognostic index which reliably selects a group of patients (65%) with at least a 70% chance of being progression free from 1 year onwards. Patients who have never achieved a CR and have received three or more chemotherapy regimens do not benefit from high-dose chemotherapy as used in this study.


British Journal of Cancer (1996) 73, 1272–1277
© 1996 Stockton Press All rights reserved 0007–0920/96 $12.00

### High-dose chemotherapy and autologous bone marrow transplant in relapsed Hodgkin's disease – a pragmatic prognostic index

MER O'Brien, S Milan, D Cunningham, AL Jones, M Nicolson, P Selby, T Hickish, M Hill, ME Gore and C Viner

*Cancer Research Campaign, Section of Medicine and Lymphoma Unit, The Institute of Cancer Research and The Royal Marsden Hospital, Sutton, Surrey, UK.*

**Summary** High-dose chemotherapy with autologous bone marrow transplantation is used in the treatment of relapsed or high-risk Hodgkin's disease. As prospective randomised studies have proved difficult to accrue to, current recommendations are based on the reports of large series of prospectively collected data. We have looked at the outcome of 89 patients treated in this way at a single institution and have developed an index to predict outcome. Of 89 patients, with a median age of 29 years (range 15–51 years), eight patients were in first complete remission/partial remission (CR/PR), 17 in second or later CR, 37 were responding relapses, 13 resistant relapses, 11 primary refractory and three untested relapses. Combinations of melphalan, BCNU and etoposide were given in all cases except in ten patients who received melphalan alone. The median follow-up was 43 months (range 6–77 months). A total of 24 patients were in CR at the time of autologous bone marrow transplantation (ABMT), 33 achieved CR with ABMT, 16 PR, to give a response rate to ABMT of 49/65=74% (95% CI 60–83%) with a CR rate of 51% (CI 36–62%). In a Cox's multivariate analysis the most important factors in predicting outcome after ABMT were response to treatment before entry, number of previous treatments and previous chemosensitivity. Using these factors we devised a prognostic index which reliably selects a group of patients (65%) with at least a 70% chance of being progression free from 1 year onwards. Patients who have never achieved a CR and have received three or more chemotherapy regimens do not benefit from high-dose chemotherapy as used in this study.

**Keywords:** autologous bone marrow transplantation; high-dose chemotherapy; Hodgkin's disease; prognostic index

The mortality rate of patients receiving high-dose chemotherapy with haematological support in the form of autologous bone marrow transplantation (ABMT) has improved with better patient selection, supportive medicine and the use of peripheral blood stem cells and growth factors (Peters, 1993). This procedure remains unproven in Hodgkin's disease (HD) and at present decisions are based on the results reported in one randomised trial and other retrospective series of patients treated at different stages of HD when drug resistance is either present or beginning to appear.

From the observation that patients with relapsed disease when treated with conventional chemotherapy have a progression-free survival (PFS) of around 30% and a median survival of 12 months, it has become accepted that fit patients with HD who have a first remission of <12 months' duration, those who fail to achieve a complete remission (CR) with satisfactory induction therapy including an anthracycline, or those patients who are in second or subsequent relapse, or high-risk patients in first relapse, should be treated in this way (Goldstone and McMillan, 1993; Longo *et al.*, 1992).

Lohri *et al.* (1991) described a group of 80 patients after first relapse HD treated by four different treatment regimens including one group of 16 patients who received high-dose chemotherapy with ABMT. Freedom from second relapse was the end point and was similar in all groups. More recently the Vancouver group have reported their experience with high-dose treatment in a group of HD patients after first relapse from initial complete remission and compared the outcome with that in patients treated with conventional chemotherapy (Reece *et al.*, 1994). The authors report a 64% PFS for the group of 58 patients which is better than results with conventional chemotherapy, e.g. the Milan group reported a PFS of 46% in patients with initial remissions of more than a year (Bonfante *et al.*, 1993).

There is one randomised trial comparing high-dose chemotherapy [BCNU, etoposide, cytarabine and melphalan (BEAM)] with ABMT with a less intensive regimen (miniBEAM) in patients with active HD who had failed conventional chemotherapy. The numbers were small but did show a significant event-free survival advantage (53% *vs* 10%) at a median follow-up of 34 months ($P=0.025$) in favour of BEAM plus ABMT. There was no difference in overall survival (Linch *et al.*, 1993). The trial demonstrates the difficulty in recruiting to a trial in which one of the treatment modalities, although unproven, is seen to be the best chance of long-term survival. A few years into this randomised study, patients were refusing to be randomised and requested high-dose treatment.

We report our experience at a single centre treating relapsed or non-responding HD, the most common category of patients seen being the sensitive relapses with a varying number in each other subgroup. With long follow-up we report both long- and short-term toxicity, response rates and prognostic factors to help predict which patients will have a long PFS

### Patients and methods

A group of 89 unselected patients treated at a single institution with high-dose chemotherapy and bone marrow transplant between 1 October 1985 and 1 March 1992 is presented.

### *Patients and previous treatment*

Characteristics at diagnosis and at the time of high-dose chemotherapy and ABMT are shown in Table I. Data on lactate dehydrogenase (LDH) levels were not available in a large enough percentage of patients to be included in the

Correspondence: D Cunningham, Section of Medicine, The Royal Marsden Hospital NHS Trust, Downs Rd, Sutton, Surrey, SM2 5PT, UK
Received 7 August 1995; revised 13 December 1995; accepted 14 December 1995

analysis. The median follow-up of survivors was 43 months (range 6–77 months). At the time of ABMT, the median age was 29 years (range 15–51 years). The median time from diagnosis until ABMT was 2.5 years (range 4.3 months–14 years) There were 58 patients who were treated at hospitals and were referred in remission for high-dose consolidation therapy. The first treatment regimen given at the onset of disease was ChlVPP, 37 patients; MOPP, four patients; LOPP, nine patients; VEEP, eight patients; other anthracycline combination, 26 patients, and radiotherapy alone, five patients. Before high-dose therapy all patients had received previous chemotherapy with a median of two regimens per patient. There were 51/89 patients who had received radiotherapy with 37/51 receiving radiotherapy to the mediastinum. Bleomycin had been given to 39/89 patients during previous chemotherapy.

Seven patients in first CR were all patients with advanced stage disease (IIIA, two patients; IIIB, two patients; and IVB, three patients) who had required at least two treatment regimens to obtain their first CR. Thus, 24 patients were in complete remission (CR) at the time of high-dose treatment. There were 41 patients in partial remission (PR) after the most recent chemotherapy, i.e. before high-dose treatment, 12 had no response (NR), nine had progressive disease (PD) and three were untested. It was not possible, retrospectively, to define patients who had a CR unconfirmed (CRu), as this is a recently introduced term of response. According to the known categories described by Philip *et al.* (1987) in relapsed non-Hodgkin's lymphoma at the time of ABMT, eight patients were in first CR/PR, 17 in second or later CR, 24 were responding relapses, 13 resistant relapses, 11 primary refractory and three untested relapses (Table II).

*Conditioning regimens*

Melphalan as a single agent (180–220 mg $m^{-2}$) was used in the first phase of the study in ten patients. BCNU (300–600 mg $m^{-2}$) was then added to the melphalan (80–140 mg $m^{-2}$) and given to 11 patients and then etoposide (300 mg $m^{-2}$) was added to the combination (MBE) and given to 61 patients. Melphalan (140 mg $m^{-2}$) and etoposide (1200 mg $m^{-2}$) without BCNU was given to seven patients who had poor pulmonary function tests. Melphalan was given with intravenous hydration and forced diuresis and when used as a single agent fresh bone marrow was returned 6 h after infusion of the drug. Combinations of drug were given as per standard protocols with cryopreserved bone marrow (Harding *et al.*, 1992).

**Table I** Patient characteristics ($n$ = 89)

| | |
|---|---|
| Gender | |
| Male | 54 |
| Female | 35 |
| Stage at diagnosis | |
| I | 2 |
| II | 24 |
| III | 29 |
| IV | 34 |
| Symptoms at diagnosis | |
| A | 35 |
| B | 54 |
| At ABMT | |
| Age (years) | |
| Median | 29 |
| Range | 15-51 |
| Symptoms | |
| A | 71 |
| B | 18 |
| Nodal disease | |
| Yes | 59 |
| No | 30 |
| Extranodal disease | |
| Yes | 31 |
| No | 58 |
| CR ever | |
| Yes | 65 |
| No | 24 |
| Number of previous chemotheropy regimens | |
| 1 | 3 |
| 2 | 44 |
| 3 | 28 |
| 4 | 8 |
| 5 | 6 |

*Statistical methods*

The unit policy was to treat to maximum response as defined by UICC criteria (Miller *et al.*, 1981). Kaplan–Meier actuarial survival and progression-free survival were calculated for all patients. Progression rather than survival was chosen for the Cox analysis because there were a large number of late deaths owing to factors other than HD in this group of patients. The factors causing these deaths are not the same as the factors influencing death from HD, and as some patients who died did not have relapsed HD it was decided that the strongest model for predicting the effect of ABMT on the disease process would be obtained by examining the factors influencing progression.

The three untested relapses were not included in the Cox's analysis and the analysis was repeated with and without the patients in first CR ($n$=7) and first PR ($n$=1). Neither of these omissions significantly changed any results.

An early treatment-related death was defined as a death within 2 months of ABMT. After the initial 2 month transplant period subsequent events have been termed late toxicities.

### Results

*Response rate*

A total of 24 patients were in CR at the time of ABMT, 33 achieved CR with ABMT, 16 PR, 16 NR and three early treatment deaths (two of these patients were in CR before ABMT) to give a response rate to ABMT of 49/65=74% (95% CI 60–83%) with a CR rate of 33/65=51% (CI 36–62%). A total of 57/89=64% of patients were therefore in CR at the end of the procedure.

*Primary refractory disease and resistant relapses (Table II)*

In all, 6/11 patients had a complete remission. Two of these patients developed pneumonitis and died without relapsed disease, both had a BCNU dose of 600 mg $m^{-2}$, one had mediastinal radiotherapy and both had received two previous chemotherapy regimens. One patient in CR died of infection having had four previous treatment regimens, two patients have relapsed and only one remains in remission 56 months later.

In the group of resistant relapses, no CR were documented and 6/13 patients achieved a PR, 4/6 have died of progressive disease, one is alive at 20 months in continued PR and one

**Table II** Outcome by status at ABMT

| | | *Outcome after ABMT* | | | |
|---|---|---|---|---|---|
| *Status at ABMT* | *Already CR* | *CR* | *PR* | *NR* | *Total* |
| First CR/PR | 7 | 8* | 0 | 0 | 8 |
| Subsequent CR | 17 | 17* | 0 | 0 | 17 |
| Responding relapse | 0 | 24 | 5 | 8 | 37 |
| Resistant relapse | 0 | 0 | 6 | 7 | 13 |
| Primary refractory | 0 | 6 | 4* | 1 | 11 |
| Untested relapse | 0 | 2 | 1 | 0 | 3 |

*Patient within group who had treatment-related death, i.e. within 2 months of treatment.

went from a PR to a CR with mantle radiotherapy and is alive and well 43 months on. A patient with NR is still alive at 16 months.

*Deaths and early/late toxicities (Table III)*

Three toxic deaths occurred early (within 2 months of the transplant), one with acute renal failure and the other two with multisystem failure probably owing to sepsis. Two of these three patients were in complete remission before transplant. There have been eight treatment-related deaths in patients in remission (seven CR, one PR), one patient developed fevers 3 months after transplant and died of 'septicaemia' in another hospital, no post-mortem was obtained and this patient was classified as in continued remission. One patient assessed as in PR after high-dose treatment died of cryptococcus meningitis 8 months after treatment; no evidence of HD was found at post-mortem. The details of the six patients who died of lung toxicity will be the subject of a separate publication.

There were two cases of leukaemia in the remission group and as these patients were heavily pretreated this is not considered a direct toxicity of the high-dose therapy. One patient with progressive disease going into high-dose treatment died of a protein-losing enteropathy 2.5 months after treatment and unfortunately no post-mortem was permitted. One patient with a background of a depressive personality, in remission, committed suicide 4.5 years after high-dose treatment. Thus the late toxic mortality rate overall is 8/86 = 9%, or including the three early toxic deaths 11/89 = 12% and in the complete remission patients the late toxic mortality rate was 7/55 = 13%.

*Second malignancies (Table III)*

There were three deaths from second malignancies: one acute myeloid leukaemia, one myelodysplastic syndrome with subsequent leukaemia, in two patients in remission at 4 and 6 years, and one high-grade non-Hodgkin's lymphoma at 3 years in a patient with active HD. A fourth patient, aged 38 years, died of active HD but had also developed a squamous carcinoma of the oral mucosa.

*Survival*

All patients are included in the survival analysis. Survival overall is shown in Figure 1 with a 5 year survival of 41%.

*Progression-free survival (PFS) and time to progression*

The median PFS overall is 17 months with 40% alive and progression-free at 5 years (Figure 2). However, for those in CR at the time of ABMT or afterwards the probability of

**Figure 1** Overall survival ($n = 89$).

**Figure 2** Overall progression-free survival ($n = 89$).

**Figure 3** Progression-free survival by response to chemotherapy before high-dose treatment.

**Table III** Late toxic deaths

| *Outcome following ABMT* | *Total* | *Deaths* | *PD* | *Active HD*[a] | *In remission* |
|---|---|---|---|---|---|
| CR | 55 (62%) | 19 | 9 | 2[b] | 8[c] |
| PR | 15 | 12 | 7[d] | 3[e] | 2[f] |
| NR | 16 | 12 | 9[g] | 3[h] | |

[a]Plus other cause of death. [b]One pneumonitis, one multifocal encephalopathy. [c]Six pneumonitis, one infection: 'septicaemia', no post mortem, 'one leukaemia'; [d]One squamous cell cancer of the head and neck. [e]Three infections: fungal, TB, bronchopneumonia. [f]One infection: cryptococcus meningitis; one leukaemia. [g]One protein-losing enteropathy. [h]One high-grade NHI.

remaining progression-free is 75% at 20 months and this is maintained up to 5 years (Figure 3). Removing the patients with first CR does not change this graph. The PR graph plateaus after 42 months at 60% and the NR/PD graph plateaus after 15 months at 33%. There is no significant difference between the group of NR and PD ($P=0.27$).

*Univariate analysis*

A total of 21 factors were looked at in univariate analysis for influence on progression-free survival and survival at 5 years. Performance status at the time of ABMT was not always documented and therefore the accuracy of a retrospective assessment from the case notes was considered unacceptable. The following showed no significant influence on PFS or survival: gender, histology, age ($\leqslant$25 or>25 years), stage at ABMT, symptoms ever (A/B), symptoms at ABMT, extranodal disease at ABMT, bulky disease at ABMT, category at ABMT (first CR, second CR, primary refractory, responding relapse, resistant relapse), CR ever, number of CRs ($\leqslant 1$, $>1$), previous radiotherapy, number of previous chemotherapy regimens ($<3$, $\geqslant 3$), total number of courses of previous chemotherapies ($>13$, $\leqslant 13$), length of longest remission ($>2$ years, $\leqslant 2$ years), previous bleomycin, ABMT conditioning regimen, dose of BCNU ($\leqslant 400$, $>400$), albumin at ABMT in g $dl^{-1}$ ($<35$, $>35$). The outcome following the most recent treatment before high-dose treatment (with or without the three untested) was the most significant factor for both progression-free survival (chi-squared test=9.69, $P<0.025$, Figure 3) and overall survival ($P=0.02$). Treatment/response history in two groups as follows: $<3$ regimens or previous CR as one group or $\geqslant 3$ regimens but never CR as a second group, was also significant for progression-free survival and survival ($P=0.004$ and $P=0.01$ respectively).

**Table IV** Multivariate analysis

| *Outcome following most recent chemotherapy before high-dose treatment* | *Hazard ratio (for PFS)* |
|---|---|
| CR | 1 |
| PR | 1.87 |
| NR or progression | 3.74 |
| <3 regimens or previous CR | 1 |
| ⩾3 regimens and no CR | 3.19 |
| Responding relapse | 1.71 |
| Primary refractory or resistant relapse | 3.42 |

**Figure 4** Progression-free treatment/response to chemotherapy proir to high-dose treatment.

*Multivariate analysis (Table IV)*

A Cox's multivariate analysis produced a model in which response to the chemotherapy regimen before ABMT and treatment/response history were the factors which predicted long-term freedom from progression with this procedure (Table IV, Figures 3 and 4). No other additional factor improved the fit. Putting these two groups together produced four Cox groups A–D (A, CR to most recent treatment; B, PR to most recent treatment, CR previously or $<3$ regimens; C, NR/PD to most recent treatment, CR previously or $<3$ regimens; D, never CR and $\geqslant 3$ regimens) to give a predictive model shown in Figure 5. This model divides the patient population broadly into a group of 56/86 (65%) patients (Cox group A+B, i.e. CR or PR to most recent treatment and a CR previously or $<3$ previous treatment regimens) with at least a 70% chance of being progression free from 1 year onwards and the other groups (Cox group C, D) with a poorer prognosis (chi-square=12.46, $P<0.01$). If response to the last chemotherapy was excluded from the model, then the category at ABMT (e.g. sensitive relapse etc.) produced a model which was nearly as good as the model constructed using sensitivity to the previous chemotherapy regimen (Table IV, Figure 6).

**Figure 5** Progression-free survival by response to ABMT by Cox group from multivariate analysis.

**Figure 6** Progression-free survival using sensitivity classification.

**Table V** Studies of high-dose chemotherapy in Hodgkin's disease

| | No. of patients | Mortality (%) | CR rate (%) | Median follow-up-months | PFS overall (%) (CR %) 24–36 months | 48 months | 60 months |
|---|---|---|---|---|---|---|---|
| Carella *et al.* (1988) | 50 | 7 | 48 | 24 | 45 | 45 | |
| Jagannath *et al.* (1989) | 61 | 7 | 47 | 24 | 40 (77) | | |
| Phillips *et al.* (1989) | 26 | 23 | 69 | 54 | | 38 (39) | |
| Jones *et al.* (1990) | 28 | 21 | 64 | 26 | 51 (65) | | |
| Reece *et al.* (1991) | 56 | 21 | 80 | 42 | | | 47 |
| Gianni *et al.* (1993) | 25 | 0* | 72 | 67 | | | 48 (78) |
| Tourani *et al.* (1992) | 39 | 10 | 79 | | | | 48 |
| Chopra *et al.* (1993) | 155 | 10 | 34 | 24 | | | 50 |
| Crump *et al.* (1993) | 73 | 10 | 75 | 30 | | 39 (68) | |
| Goldstone and McMillan (1993) | 947 | 29 | 34 | 37 | | | 35 (40) |
| Vose *et al.* (1992) | 70 | 11 | 59 | 36 | 51 (22) | | |
| Yalahom *et al.* (1993) | 47 | 17 | 74 | 40 | 52 (80) | 50 | 50 (80) |
| Rapoport *et al.* (1993) | 47 | 25 | 24 | 24 | 49 (70) | | |
| Anderson *et al.* (1993) | 68 | | | | | | 18 |
| Bierman *et al.* (1993) | 128 | 17 | 48 | 77 | | | 25 |
| Royal Marsden Hospital, (1995) | 89 | 22 | 67 | 43 | 48 (75) | 45 | 41 (75) |

Only includes studies with at least 24 patients and a median follow up of >24 months. * + GCSF; + PBSC.

### Discussion

There have been two reports from this centre on the use of high-dose treatment in Hodgkin's disease (Zulian *et al.*, 1989; Harding *et al.*, 1992). For this analysis the database has been updated and status of disease and response to previous treatment and high-dose treatment verified by two physicians. In addition, some patients in the earlier report were treated with single-agent melphalan at suboptimal doses (< 140 mg $m^{-2}$) and are now excluded. There are now 89 patients with a median follow-up of survivors of 43 months (range 6–77 months), which means that this is one of the larger series in the literature and meaningful interpretation can be given to both the survival and progression-free survival data and to the results of a multivariate analysis on the prediction of response to this treatment.

For patients who achieved a CR before ABMT, the plateau on the graph is at 75% with no late relapses. For PRs before high-dose chemotherapy, the plateau occurs after 45 months and remains at 60%. There is also a plateau on the graph for the group of NR/PD after 15 months and remaining at 33%. At this point in our series we have three cases of second malignancies that have resulted in death. Our acute toxicities were due in most cases to lung disease which can now be predicted and avoided in many cases by reduction of the BCNU dose and the early use of steroids.

To date, the 'category' at ABMT as originally described for non-Hodgkin's lymphoma, e.g. resistant relapses etc., has been the gold standard for response assessment. This categorisation does produce a model very similar to that obtained using response to last chemotherapy but only when response to last chemotherapy has been excluded from the analysis (Figure 6). However, retrospective assessment of response is difficult, with the possible exception of a CR. Our prognostic model has been constructed using the hard end points of previous treatment, namely the attainment of a CR ever, the number of previous chemotherapy regimens and the response to the chemotherapy before high-dose therapy. Using this approach we have been able reliably to select the group of patients for whom high-dose chemotherapy will be most successful (hazard ratio = 1) and identify poor risk cases. This prognostic index remains unproven but could easily be applied to bone marrow transplantation registry data.

Table V summarises the larger reported series of similar patients treated with high-dose chemotherapy with a median follow-up of at least 24 months and at least 25 patients in each series. This is an attempt to compare our results with other groups. Jones *et al.* (1990) reported their series of 50 patients which included 21 patients treated with an allograft and found that a complete or partial remission at the time of transplant was not a significant prognastic factor, but the presence of sensitive disease at the time of transplantation was a favourable prognostic sign for surviving event-free. The efficacy of high-dose chemotherapy with bone marrow rescue has been shown to be greater when there is some degree of sensitivity to the therapy (Lohri *et al.*, 1991; Carella *et al.*, 1988; Jagannath *et al.*, 1989) Lohri *et al.* (1991) also reported a very strong negative impact on outcome in patients with stage IV disease at presentation, recurrence within a year of primary treatment or presence of B symptoms ar recurrence. We did not find any of these factors to be significant in our study as most have been employed to predict response to second line therapy whereas most of our patients were at different stages in their disease, usually much later when the duration of first response may not have been relevant. In addition, like so many other high-dose experiences, the group is made up of a number of subtypes of disease and the paucity of numbers in each group makes any interpretation meaningless; a meta-analysis may help in the subgroup analysis.

This prognostic index should now be applied to a large series of patients with Hodgkin's disease who have received high-dose treatment.

**Acknowledgements**
MO'B, SM, MN, TH, MH were all supported by the UK Cancer Research Campaign.

### References

ANDERSON JE, LITZOW MR, APPELBAUM FR, SCHOCH G, FISHER LD, BUCKNER CD, PETERSEN FB, CRAWFORD SW, PRESS OW AND SANDERS JE. (1993). Allogeneic, syngeneic, and autologous marrow transplantation for Hodgkin's disease: the 21-year Seattle experience. *J. Clin. Oncol.*, **11**, 2342–2350.

BIERMAN PJ, BAGIN RG, JAGANNATH S, VOSE JM, SPITZER G, KESSINGER A, DICKE KA AND ARMITAGE JO. (1993). High dose chemotherapy, followed by autologous hematopoietic rescue in Hodgkin's disease: long term follow-up in 128 patients. *Ann. Oncol.*, **4**, 767–773.

BONFANTE V, SANTORO A, DEVIZZI L, BALZAROTTI M, VIVANI S, ZANINI M, VALAGUSSA P AND BONADONNA G. (1993). Outcome of patients with Hodgkin's disease relapsing after alternating MOPP/ABVD (abstract). *Proc. Am. Soc. Clin. Oncol.*, **12**, 364.

CARELLA AM, CONGIU AM, GAOZZA E, MAZZA P, RICCI P, VISANI G, MELONI G, CIMINO G, MANGONI L AND COSER P. (1988). High-dose chemotherapy with autologous bone marrow transplantation in 50 advanced resistant Hodgkin's disease patients: an Italian Study Group report. *J. Clin. Oncol.*, **6**, 1411–1416.

CHOPRA R, MCMILLAN AK, LINCH DC, YUKLEA S, TAGHIPOUR G, PEARCE R, PATTERSON KG AND GOLDSTONE AH. (1993). The place of high-dose BEAM therapy and autologous bone marrow transplantation in poor risk Hodgkin's disease. *Blood*, **81**, 1137–1145.

CRUMP M, SMITH AM, BRANDWEIN J, COUTURE F, SHERRET H, SUTTON DM, SCOTT JG, MCCRAE J, MURRAY C AND PANTALONY D. (1993). High-dose etoposide and melphalan and autologous bone marrow transplantation for patients with advanced Hodgkin's disease: importance of disease status at transplant. *J. Clin. Oncol.*, **11**, 704–711.

GIANNI AM, SIENA S, BREGNI M, LOMBARDI F, GANDOLA L, DI NICOLA M, MAGNI M, PECCATORI F, VALAGUSSA P AND BONADONNA G. (1993). High-dose sequential chemo-radiotherapy with peripheral blood progenitor cell support for relapses or refractory Hodgkin's disease. *Ann. Oncol.*, **4**, 889–891.

GOLDSTONE AH AND MCMILLAN AK. (1993). The place of high-dose therapy with haemopoietic stem cell transplantation in relapsed and refractory Hodgkin's disease. *Ann. Oncol.*, **4** (suppl.1), S21–27.

HARDING M, SELBY P, GORE M, PERREN T, TRELEAVAN J, MANSI J, ZULIAN G, MILAN S, MELDRUM M AND VINER C. (1992). High-dose chemotherapy and autologous bone marrow transplantation for relapsed and refractory Hodgkin's disease. *Eur. J. Cancer*, **28A**, 1396–1400.

JAGANNATH S, ARMITAGE JO, DICKE KA, TUCKER SL, VELASQUEZ WS, SMITH K, VAUGHAN WP, KESSINGER A, HORWITZ LJ AND HAGEMEISTER FB. (1989). Prognostic factors for response and survival after high-dose cyclophosphamide, carmustine, and etoposide with autologous bone marrow transplantation for relapsed Hodgkin's disease. *J. Clin. Oncol.*, **7**, 179–185.

JONES RJ, PIANTADOSI S, MANN RB, AMBINDER RF, SEIFTER EJ, VRIESENDORP HM, ABELOFF MD, BURNS WH, MAY WS AND ROWLEY SD. (1990). High-dose cytotoxic therapy and bone marrow transplantation for relapsed Hodgkin's disease. *J. Clin. Oncol.*, **8**, 527–537.

LINCH DC, WINFIELD D, GOLDSTONE AH, MOIR D, HANCOCK B, MCMILLAN A, CHOPRA R, MILLIGAN D AND HUDSON GV. (1993). Dose intensification with autologous bone marrow transplantation in relapsed and resistant Hodgkin's disease: results of a BNLI randomised trial. *Lancet*, **341**, 1051–1054.

LOHRI A, BARNETT M, FAIREY RN, O'REILLY SE, PHILLIPS GL, REECE D, VOSS N AND CONNORS JM. (1991). Outcome of treatment of first relapse of Hodgkin's disease after primary chemotherapy: identification of risk factors from the British Columbia experience 1970–1988. *Blood*, **77**, 2292–2298.

LONGO DL, DUFFEY PL, YOUNG RC, HUBBARD SM, IDHE DC, GLATSTEIN E, PHARES JC, JAFFE ES, URBA WJ AND DEVITA JR VT. (1992). Conventional-dose salvage combination chemotherapy in patients relapsing with Hodgkin's disease after combination chemotherapy: the low probability of cure. *J. Clin. Oncol.*, **10**, 210–218.

MILLER AB, HOOGSTRATEN B, STAQUET M AND WINGLER A. (1981). Reporting results of cancer treatment. *Cancer*, **47**, 207–214.

PETERS WP. (1993). Evolving concepts in dose-intensive chemotherapy for node-positive breast cancer. *Adv. Oncol.*, **8**, 17–25.

PHILIP T, ARMITAGE JO, SPITZER G, CHAUVIN F, JAGANNATH S, CAHN JY, COLOMBAT P, GOLDSTONE AH, GORIN NC AND FLESH M. (1987). High-dose therapy and autologous bone marrow transplantation after failure of conventional chemotherapy in adults with intermediate grade or high-grade non-Hodgkin's lymphoma. *N. Engl. J. Med.*, **316**, 1493–1498.

PHILLIPS GL, WOLFF SN, HERZIG RH, LAZARUS HM, FAY JW, LIN HS, SHINA DC, GLASGOW GP, GRIFFIN RC AND LAMB CW. (1989). Treatment of progressive Hodgkin's disease with intensive chemoradiotherapy and autologous bone marrow transplantation. *Blood*, **73**, 2086–2092.

RAPOPORT AP, ROWE JM, KOUIDES PA, DUERST RA, ABBOUD CN, LIESVELD JL, PACKMAN CH, EBERLY S, SHERMAN M AND TANNER MA. (1993). One hundred autotransplants for relapsed or refractory Hodgkin's disease and lymphoma: value of pretransplant disease status for predicting outcome. *J. Clin. Oncol.*, **11**, 2351–2361.

REECE DE, BARNETT MJ, CONNORS JM, FAIREY RN, FAY JW, GREER JP, HERZIG GP, HERZIG RH, KLINGEMANN HG AND LEMAISTRE CF. (1991). Intensive chemotherapy with cyclophosphamide, carmustine, and etoposide followed by autologous bone marrow transplantation for relapsed Hodgkin's disease. *J. Clin. Oncol.*, **9**, 1871–1879.

REECE DE, CONNORS JM, SPINELLI JJ, BARNETT MJ, FAIREY RN, KLINGEMANN HG, NANTEL SH, O'REILLY S, SHEPERD JD AND SUTHERLAND HJ. (1994). Intensive therapy with cyclophosphamide, carmustine, etoposide +/– cisplatin and autologous bone marrow transplantation for Hodgkin's disease in first relapse after combination chemotherapy. *Blood*, **83**, 1193–1199.

TOURANI JM, LEVY R, COLONNA P, DESABLENS B, LEPRISE PY, GHILHOT F, BRAHIMI S, BELHANI M, IFRAH N AND SENSEBE L. (1992). High-dose salvage chemotherapy without bone marrow transplantation for adult patients with refractory Hodgkin's disease. *J. Clin. Oncol.*, **10**, 1086–1094.

VOSE JM, PHILLIPS GL AND ARMITAGE JO. (1992). Autologous bone marrow transplantation for Hodgkin's disease, In *High-dose Cancer Therapy: Pharmacology, Hematopoiesis, Stem Cells*. Armitage JO, Antman KH (eds) pp. 651–661. Williams & Wilkins: Baltimore.

YALAHOM J, GULATI SC, TOIA M, MASLAK P, MCCARRON EG, O'BRIEN PJ, PORTLOCK CS, STRAUS DJ, PHILLIPS J AND FUKS Z. (1993). Accelerated hyperfractionated total-lymphoid irradiation, high-dose chemotherapy, and autologous bone marrow transplantation for refractory and relapsing patients with Hodgkin's disease. *J. Clin. Oncol.*, **11**, 1062–1070.

ZULIAN GB, SELBY P,MILAN S, NANDI A, GORE M, FORGESON G, PERREN TJ AND MCELWAIN TJ. (1989). High dose melphalan, BCNU, and etoposide with autologous bone marrow transplantation for Hodgkin's disease. *Br. J. Cancer*, **59**, 631–635.